# Influence of Fuel and Technology on Particle Emissions from Biomass Cookstoves—Detailed Characterization of Physical and Chemical Properties

**DOI:** 10.1021/acsomega.4c07785

**Published:** 2025-01-29

**Authors:** Robert Lindgren, Natxo García-López, Karin Lovén, Lisa Lundin, Joakim Pagels, Christoffer Boman

**Affiliations:** † Thermochemical Energy Conversion Laboratory, Department of Applied Physics and Electronics, Umeå University, SE-90187 Umeå, Sweden; ‡ Ergonomics and Aerosol Technology, Lund University, LTH, SE-22100 Lund, Sweden; § Department of Chemistry, Umeå University, SE-90187 Umeå, Sweden

## Abstract

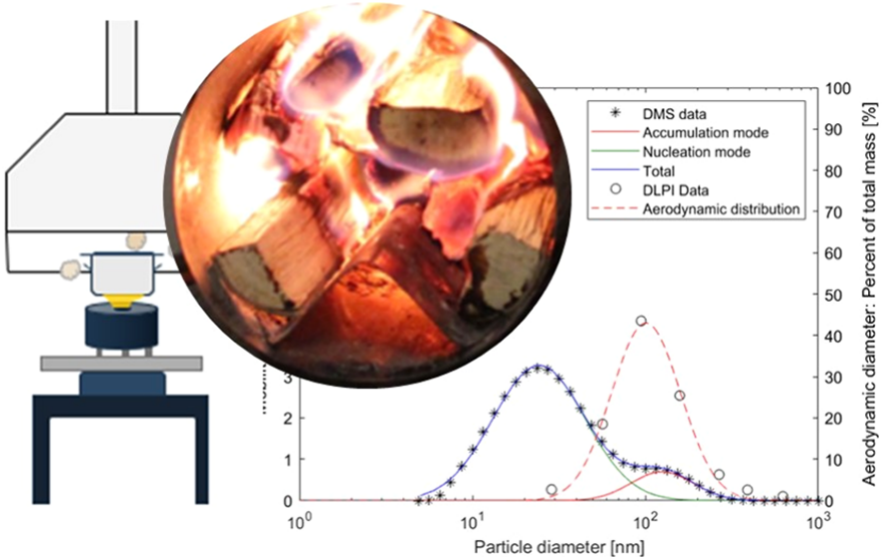

Globally, 3 billion people rely on solid biomass fuel for their everyday cooking, most often using inefficient cooking practices, leading to high exposure levels of household air pollution. This is subsequently associated with negative health and climate impact. Further, the inefficient use of biomass fuels applies pressure on natural forests, resulting in deforestation, loss of biodiversity, and soil degradation. Improved cookstove technologies and biomass fuels are being promoted to mitigate these issues. However, limited knowledge exists about how the interaction between stove technology and new fuels affects the physical and chemical properties of particulate emissions. In this study, the emission performance of four cookstove technologies in combination with five fuels was evaluated in a laboratory setup, applying a modified water boiling test with a hood dilution system for flue gas sampling. Filter sampling was applied to determine the emissions of fine particulate matter (PM_1_) and for subsequent analysis of polycyclic aromatic compounds (PAC), organic- and elemental carbon, and inorganic composition. Particle mass size distribution was determined by using a 13-stage low-pressure cascade impactor. Online instruments were used to determine gaseous emissions (e.g., CO, CH_4_, and BTX) as well as particle number size distribution. The results show that both the stove design and fuel properties influence the total emissions as well as the physiochemical PM characteristics. It was further seen that the impact of fuel on the PM properties did not translate linearly among the different stove technologies. This implies that each stove should be tested with various fuels to determine both the total emissions and fuel suitability.

## 1 Introduction

Every day, 3 billion people are exposed to harmful household air pollution (HAP), attributed to gaseous pollutants and aerosol particles emitted from cooking with solid fuels, mainly in low- and middle-income countries (LMIC).^1,2^ The traditional and still most common cooking practice is the so-called 3-stone open fire using wood logs/sticks, which has low fuel efficiency and generates high emissions of hazardous air pollutants, e.g., CO, hydrocarbons, and soot. Particulate matter (PM) in ambient air is today considered to be the most problematic pollution component with respect to public health concerns,^3^ and the fine (submicron) PM fraction is of particular importance regarding biomass combustion-related air pollution.^4,5^ Drivers of toxicity related to exposure to PM derived from biomass combustion include, e.g., soot surface area, metals, and polycyclic aromatic hydrocarbons (PAH).^4^ Furthermore, it has been shown that the toxicity of PAH is specifically enhanced when they are adsorbed onto soot surfaces.^6^ Indoor exposure to these emissions in LMIC is associated with severe health degeneration through diseases like acute lower respiratory infections, chronic obstructive pulmonary disease (COPD), lung cancer, and cardiovascular diseases.^7^ It has been estimated that HAP from indoor cooking with solid fuels is associated with 3.2 million premature deaths, annually,^8^ with women and children being the most affected.^9,10^ In addition, emissions of soot, referred to as black carbon (BC), affect the global climate directly by absorbing incoming solar radiation,^11^ and are considered to be one of the most important short-lived climate forcers (SLCF).^12^ The extensive use of solid biomass fuels in inefficient stoves in LMIC is also associated with severe deforestation and land degradation, resulting in several negative environmental impacts such as soil erosion, flash floods, droughts, and fuel shortages.^13^

Extensive efforts are in progress to mitigate these issues by promoting so-called clean cooking through the introduction of new solutions such as electricity and gas stoves, as well as improved biomass stoves and upgraded fuels. However, using electric stoves for household cooking is still a far vision for millions of people due to considerable challenges associated with the production, distribution, and grid capacity of new power supply systems. In some regions, like sub-Saharan Africa (SSA), the situation is extra prominent and the efforts to increase access to electricity are often outpaced by population growth.^14^ Another route for the transition into cleaner cooking is via liquefied petroleum gas (LPG) stoves. Although LPG stoves are “clean” regarding direct emissions and HAP,^15,16^ this energy source is fossil-based, global gas market dependent, and for most households still unaffordable.^17^ This suggests that extensive reliance on solid biomass fuels worldwide, not at least in SSA, will continue in the foreseeable future.

The potential for production and supply of local and sustainable biomass fuels for energy purposes in many African and Asian LMIC is significant, and an increasing interest has been seen for upgraded biomass feedstocks, e.g., pellets and briquettes of locally available wood resources and/or agro-industrial residues.^18^ It has, for example, been estimated by Carvalho et al. that a transition toward a sustainable bioenergy strategy including clean cooking in combination with the utilization of locally available fuel value chains, may reduce the overall environmental impact by up to 80%.^19^ Agroforestry, where trees and crops are combined,^20^ has shown great potential to contribute to such bioenergy strategies and is considered a key measure to tackle interlinked challenges related to energy, poverty, food, water, climate, land use, and biodiversity.^21,22^ In addition, an increased use of agricultural residues for energy in SSA, in sustainable and modern bioenergy transitions, would also reduce deforestation and utilize currently underutilized biomass resources.^23,24^ However, by introducing new stove technologies in combination with new biomass feedstocks, the combustion conditions will vary, and consequently so will the emission performance, and the particle properties.^25^ The need for science-based knowledge and decision making, derived from both lab- and field emission studies, has therefore been highlighted to support the testing procedures, policy development, and technological implementations of new cooking solutions.^26,27^

Previous research on residential biomass combustion for heating purposes, has shown that the PM emissions are dominated by submicron particles (PM_1_) constituted of a complex and variable mixture of primarily three classes of fine particles, i.e., organic (spherical) particles, soot aggregates, and inorganic salt particles, presented in a simplified model regarding their formation and properties,^4^ as later refined in Sigsgaard et al.^5^ In the perspective work by Sigsgaard et al.,^5^ an overall assessment of the health effects of biomass burning in the developed world was also presented, which emphasized the need to further quantify and mitigate the adverse health effects of small-scale wood combustion. It is well known, from the field of thermochemical conversion of biomass, that under inefficient combustion conditions, the emissions of organic matter and soot are elevated. While the organic particles derive from the degradation and pyrolysis of condensable biomass constituents, the soot is formed in the flame via thermal transformations of the primary pyrolysis products into secondary and tertiary conversion products, eventually into PAH and subsequently solid carbonaceous (soot) aggregates.^28,29^ The formation and transformation processes of organic matter and soot are complex, and their emissions are heavily dependent on specific combustion conditions, often with high time-resolved dynamics.^30^ During more efficient biomass combustion, inorganic ash particles constitute the main part of the PM, often dominated by alkali salts and trace metals that have been volatilized from the fuel under high temperatures and subsequently forming fine particles in the flue gases.^31,32^ Thus, the ash content and composition of the fuels are crucial for the formation of this kind of PM emission, with increasing relevance, for example, related to the utilization of agricultural residues for energy purposes. However, such knowledge has rarely been applied to interpret cookstove emissions.

Significant work on cookstove emissions and HAP has been undertaken, targeting the present situation and the potential mitigation effects of introducing improved stoves.^26^ A general trend with reduced emissions for standard gas and PM components has been shown when shifting from traditional 3-stone fire to improved stoves and further to more advanced stoves using upgraded biomass fuels.^25,33,34^ A limited number of studies have also addressed the specific particle properties of the emissions from different stove technologies.^35,36^ Still, a considerable variability in emission performance exists and the importance of specific stove–fuel interactions has been faintly elucidated, not at least regarding the effects on detailed physicochemical and toxicological particle properties.^26,37^ The objective of this study was therefore to determine the influence of different cookstove technologies and biomass fuel types on particulate emissions and their detailed physical and chemical particle properties. This work addresses the specific interactions of different stove–fuel combinations when using woody fuels and novel agricultural residue fuels of relevance for SSA, in different stove technologies, with a focus on health- and climate-relevant particle properties.

## 2 Materials and Methods

### 2.1 Cookstoves

In this study, four cookstoves were used, representing different technological advancements: 3-stone open fire (3S), rocket stove (RS), natural draft gasifier stove (NDG), and forced draft gasifier stove (FDG). The 3-stone open fire is the traditional way of cooking, where the cooking pot is placed onto three stones and an open fire is lit underneath. The rocket stove, defined as improved stove technology, consists of two stainless steel pipes (Ø = 100 mm) forming an *L*-shape (*L* = 180 mm horizontal, *L* = 210 mm vertical), in which the fire is enclosed, causing a draft through the stove. Both the 3S and the RS were fueled with wood logs/sticks. The two gasifier stoves are defined as advanced stove technologies and consist of two major parts. The first part is the fuel compartment, i.e., a cylindrical container with holes at the bottom for primary air to pass through the fuel bed to support the gasification process. The second part is an outer cylindrical shell, where the fuel compartment is placed. This shell controls the amount of secondary air entering the combustion through orifices placed 1–2 cm above the fuel bed. The NDG had a larger primary air inlet area through the fuel container than the FDG. In contrast to the NDG, the FDG is provided with a fan that forces the primary and secondary air through the cookstove, thus potentially attaining more controlled combustion. Both gasifier stoves were fueled with pelletized biomass fuels.

### 2.2 Fuels

The 3S and RS were fueled with small wood logs from two different tree species: casuarina and sesbania, with higher and lower density, respectively. The wood logs were cut to a triangular cross section (base and height of around 2.5 cm) and 17 cm in length. The NDG and FDG stoves were fueled with three pelletized fuels (Ø = 8 mm); softwood, commonly used in domestic pellet boilers as a “high-quality” fuel, and two agro-industrial residue fuels from Kenya, i.e., bagasse and coffee husk. To maintain a continuous fuel conversion and flame throughout the test procedure, the bagasse and coffee husk were cocombusted with softwood in a 50/50 mix, although in this study referred to as bagasse and coffee husk, respectively. The fuel composition and characteristics are shown in Table 1.

**Table 1:** Characteristics of the Biomass Fuels Used, Including Proximate and Ultimate Analysis and Composition of Ash and Trace Metal Elements

	unit	sesbania	casuarina	softwood	bagasse^a^	coffee husk^a^
ash content (at 550 °C)	wt % db	1.7	2.0	0.3	2.3	2.8
moisture content (at 105 °C)	wt %	13.2	12.5	9.8	9.35	9.3
gross heating value	MJ/kg db	18.2	18.1	19.2	18.1	18.5
proximate analysis						
volatile matter	wt % db	80.3	82.0	85.7	83.9	80.1
fixed carbon (calculated)	wt % db	18.0	16.0	14.0	13.8	17.2
ultimate analysis						
carbon (at 1050 °C)	wt % db	48.9	48.2	51.1	49.0	49.5
hydrogen (at 1050 °C)	wt % db	6.2	6.1	6.2	6.1	6.1
oxygen (calculated)	wt % db	42.8	43.2	42.3	42.5	40.9
nitrogen (at 1050 °C)	wt % db	0.3	0.4	0.1	0.2	0.7
main and trace ash-forming elements						
S	mg/kg db	445	298	68	123	629
Cl	mg/kg db	427	602	31	46	283
K	mg/kg db	6950	3100	626	1778	10413
Na	mg/kg db	53	162	5	209	31
Ca	mg/kg db	3550	6350	1210	868	2645
Mg	mg/kg db	510	580	170	239	388
Mn	mg/kg db	28	72	178	236	253
P	mg/kg db	154	480	68	133.5	534
Al	mg/kg db	121	40	21	2041	160
Si	mg/kg db	555	117	32	7666	345
Zn	mg/kg db	7	3	20	17	20

a The bagasse and coffee husk pellets were mixed with softwood pellets in a 50/50 mixture (per weight).

### 2.3 Combustion Test Procedures

**Figure 1:**
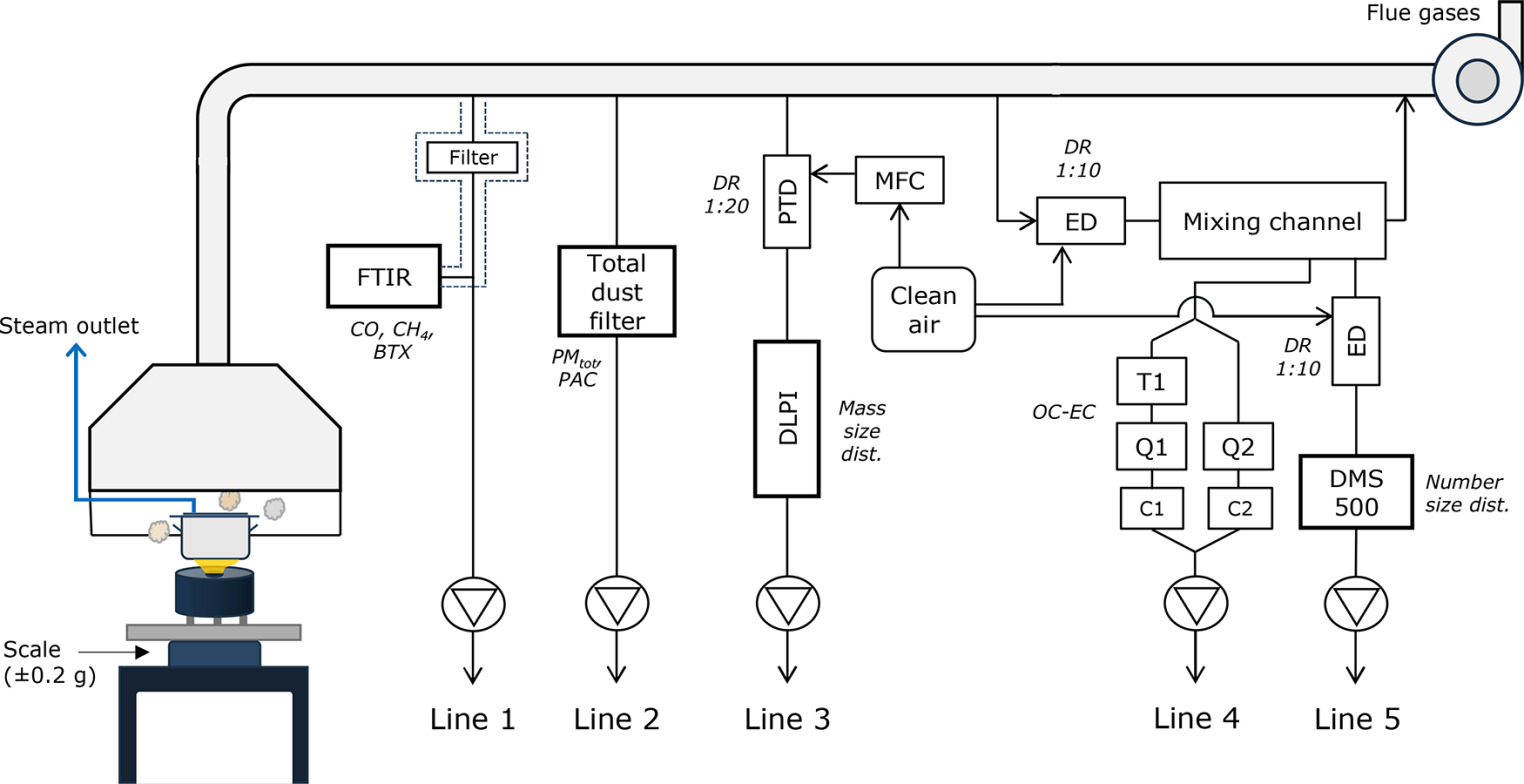
Schematic illustration of stove testing and emission sampling setup. Fourier transform infrared (FTIR) analyzer (dashed line area is heated); DMS500: differential mobility spectrometer; DLPI: Dekati low-pressure impactor; PTD: porous tube diluter; ED: ejector diluter; DR: dilution ratio; MFC: mass flow controller; T1: Teflon filter; Q1 and Q2: quartz filters; C1 and C2: critical orifices.

The cookstove combustion tests followed a modified water boiling test procedure (WBT, version 4.2.3). Two modifications were made; first, a lid connected with two tubes (Ø = 12 mm) was sealed on top of the cooking pot. The tubes conduct the steam out of the fume hood (Figure 1) to prevent the steam from interacting with the flue gases. Second, the “hot start” step of the original WBT was not followed in this study. The cookstove and the pot, including 5 kg of water, were placed under a hood, and the tests started once the fuel was ignited and ended after the water had come to boil and simmered for 45 min. For the 3S and RS, the fuel logs were added continuously throughout the run in slightly shorter intervals before the water boiled. The cookstove was placed on a scale with a resolution of ±0.2 g with a data sampling interval of 5 s. Tests for each stove–fuel combination were replicated three times. A fume hood was attached to a flue gas channel generating suction via a flue gas fan. The hood setup generated a primary dilution ratio of 1:20–1:50 depending on the burn rate of the fuels. Four separate sampling lines were connected to the flue gas channel for gas and particle measurements. A schematic illustration of the stove testing and emission sampling setup is shown in Figure 1.

### 2.4 Gas and Particle Measurements

In the first line (Line 1 in Figure 1), measurement of gaseous emissions, i.e., CO, methane, benzene, toluene, and xylene, was performed with a FTIR (Gasmet DX-4000, Gasmet Technologies Oy, Finland), connected to the flue gas channel. The secondary pyrolysis products benzene, toluene, and xylene are here referred to as BTX. In the second line (Line 2), a total dust sampling setup was used, where the particles were collected on two parallel 90 mm glass fiber filters for subsequent gravimetric analysis. The sampling probe was directed in the same direction as the flue gas flow, according to countercurrent virtual impactor inlet principles, to minimize the collection of potential coarse (>1 μm) particles (fly ash and ambient dust).^38^ Based on previous research on emissions from biomass combustion^4^ and the present sampling approach, we define the measured PM as PM_1_ in the following. The total gas volume extracted through the filters at ambient conditions was measured via a gas meter, and the particle mass concentration in the flue gases was calculated. Furthermore, the total amount of particles emitted from the stoves was calculated by multiplying the measured concentration by the total gas volume that had passed through the flue gas channel.

The aerodynamic particle mass size distribution was determined, in the third line (Line 3), through a 13-stage Dekati low-pressure impactor (DLPI) (Dekati Ltd., Finland) connected to the flue gas channel after a dilution step (DR 1:20) using a porous tube diluter (PTD). The dilution step was applied to reduce the particle concentration, thus enabling sampling throughout the entire modified water boiling test. In accordance with the total dust sampling (Line 2), the sampling probe was also directed in the same direction as the flue gas flow.

Finally, a combined fourth (Line 4) and fifth line (Line 5) was used, with a countercurrent sampling flow, followed by initial dilution (DR 1:10) with an ejector diluter (Dekati Ltd., Finland) and a subsequent mixing channel. In the fourth line, a partial flow was extracted for PM carbon fractionation analysis into organic carbon (OC) and elemental carbon (EC). An additional ejector dilution step (DR 1:10) (Dekati Ltd., Finland) was used in the fifth line (Line 5) for further dilution before online measurement of particle mobility size distribution and number concentration (5–1000 nm), using a differential mobility spectrometer (DMS500, Cambustion, UK).

### 2.5 Chemical Particle Characterization

The sampling setup for particulate carbon fractionation (organic and elemental carbon analysis) consisted of two parallel filter lines with equal flows, following standard procedures.^39^ While the first line consisted of a 47 mm PTFE filter (T1) followed by a 47 mm quartz fiber filter (Q1), the second line consisted of a single 47 mm quartz fiber filter (Q2). The Q1 and Q2 filters were analyzed for OC and EC using thermal-optical analysis (DRI Model 2001 OC/EC Carbon Analyzer, Atmoslytic, Inc., California) following the EUSAAR 2 protocol, as described by Cavalli et al.^39^ The T1 filter is expected to collect all of the solid phase particles while the semivolatile organic compounds (SVOC′s) in gas phase pass through. However, a fraction of these SVOC’s may be adsorbed on Q1, thus contributing to a positive OC artifact. According to standard protocols, the organic carbon fraction, determined from Q2, is compensated by subtracting the semivolatile material mass collected on Q1. From the OC/EC analysis the organic carbon was used to estimate the particulate organic matter (OM) by multiplication with a factor of 1.7 based on the literature,^40^ thus also estimating the mass of the H, O, and N that is part of the organic structures. Similarly, the soot mass concentration emissions were estimated by multiplying the elemental carbon with a factor of 1.1.^41^

The PTFE filter (T1) was sent to an external certified laboratory for ICP-OES analysis to determine the inorganic elemental composition in accordance with the following protocols: EN 14 775, EN 15 289, and EN 15 290. In addition, the total inorganic weight was calculated by balancing assumed cations with anions based on the ICP elemental analysis. For the cases where there was a surplus of cation-forming elements (i.e., K, Na, Ca, and Mg), the cations were stoichiometrically balanced with carbonate ions (CO_3_^2–^), and the mass calculated for K_2_CO_3_, Na_2_CO_3_, CaCO_3_, and MgCO_3_, respectively. Similarly, when there was an excess of anion-forming elements (i.e., S, P, and Cl), these were stoichiometrically balanced with H_2_O_4_, 2.5 O, and H, and the mass calculated for H_2_SO_4_, P_2_O_5_, and HCl, respectively.

Furthermore, from the total dust filters, polycyclic aromatic compounds (PAC) were analyzed, including parent polycyclic aromatic hydrocarbons (p-PAH), oxygenated PAH (O-PAH), alkylated PAH (a-PAH), and polycyclic nitrogen heterocycles (PANH). The total dust filters were extracted using pressurized liquid extraction (Dionex ASE 350, Thermo Scientific, Massachusetts) in 34 mL extraction cells with acetone/hexane (1:1) at 120 °C with three extraction cycles of 5 min each. The extracts were then purified on columns with KOH-impregnated silica gel eluted with dichloromethane, after which the eluate was evaporated, and the solvent exchanged to toluene. The samples were analyzed with gas chromatography (GC) high-resolution mass spectrometry (HRMS), using an HP 5890 GC device coupled to a Waters Autospec Ultima HRMS system. The GC assembly was equipped with a DB-5 ms capillary column (60 m × 0.25 mm × 0.25 μm; J&W Scientific, Folsom, California), and the MS was operated in electron ionization mode. Target compounds were identified by comparing GC retention data for the molecular ions in the samples and the reference standards. A lab blank was run with this set of samples. The number of individual compounds found in the blank was always below 10% of the amount found in corresponding samples, and blank corrections were therefore not performed. In total, 16 p-PAHs, 11 O-PAHs, 7 a-PAHs, and 4 PANHs were analyzed.

## 3 Results and Discussion

### 3.1 Overall Performance

The general assessment parameters for stove operation such as cooking test time, fuel consumption, and power output are summarized in Table 2. The time to complete the WBT varied between 67 and 85 min, where the shortest cooking test time was observed for the FDG using bagasse, and the longest time observed for the RS using casuarina. For both advanced cookstoves the longest test times were observed when coffee husk was used as fuel. Further, a clear trend in the relation between stove technology and fuel consumption was observed. The 3S-SES combination had the highest fuel consumption of 1786 ± 124 g, corresponding to 32.5 ± 2.3 MJ energy used, while the FDG with bagasse and coffee husk consumed roughly one-third, i.e., 601 ± 27 g, corresponding to 10.7 ± 0.5 MJ. The general trend of improved performance for the improved stoves, e.g., higher fuel efficiency, compared to the 3-stone open fire, is well in line with previous results.^34,35^ It was also observed that the fuel type had an impact on the fuel consumption for all stove technologies, apart from the FDG where there was only a minor difference between the fuels. In addition, the maximum burn rate was defined as the highest value of a 5 min moving average of the measured mass loss. However, maximum burn rates are not presented for the 3S and RS, since it is not relevant for applications with continued operation and fuel addition. For the gasifier stoves, the highest burn rates were observed when softwood and bagasse were used in the NDG stove, i.e., 5.20 ± 0.84 and 4.43 ± 0.18 kg/h, respectively. However, when the coffee husk fuel was used in the NDG, the maximum burn rate was 0.76 ± 0.04 kg/h, which is in line with the maximum burn rates seen for the FDG stove in all cases. Thus, the results from this study illustrate that the fuel properties had a certain influence on combustion performance for both 3S and RS stoves and an even more pronounced influence on the combustion performance for the NDG stove. For the FDG stove, however, no influence in fuel consumption or burn rate was seen between the three studied fuels.

**Table 2:** Fuel- and Energy-Related Parameters for the Experiments with Each Stove–Fuel Combination

	three stone (3S)			rocket stove (RS)		natural draft gasifier (NDG)			forced draft gasifier (FDG)		
	unit	sesbania	casuarina	sesbania	casuarina	softwood	bagasse	coffee husk	softwood	bagasse	coffee husk
cooking test time	[min]	73 ± 2	73 ± 1	78 ± 5	85 ± 3	73 ± 3	73 ± 1	82 ± 1	69 ± 2	67 ± 1	72 ± 1
fuel consumption	[g]	1786 ± 124	1404 ± 106	943 ± 48	883 ± 35	624 ± 43	823 ± 6	810 ± 19	621 ± 7	601 ± 5	601 ± 27
energy supplied	[MJ]	32.5 ± 2.3	24.1 ± 2.3	17.1 ± 0.9	15.2 ± 0.6	11.4 ± 0.8	14.0 ± 0.1	14.4 ± 0.3	11.3 ± 0.1	10.2 ± 0.1	10.7 ± 0.5
overall burn rate	[kg/h]	1.48 ± 0.13	1.15 ± 0.08	0.73 ± 0.07	0.62 ± 0.02	0.51 ± 0.04	0.68 ± 0.01	0.59 ± 0.03	0.54 ± 0.01	0.54 ± 0.01	0.50 ± 0.03
max burn rate	[kg/h]					5.02 ± 0.84	4.43 ± 0.18	0.76 ± 0.04	0.74 ± 0.04	0.77 ± 0.01	0.70 ± 0.02
overall power	[kW]	7.46 ± 0.68	5.50 ± 0.38	3.66 ± 0.35	2.96 ± 0.11	2.61 ± 0.21	3.19 ± 0.06	2.93 ± 0.13	2.73 ± 0.07	2.54 ± 0.05	2.48 ± 0.17
max power	[kW]					25.4 ± 4.3	20.9 ± 0.84	3.75 ± 0.18	3.73 ± 0.21	3.63 ± 0.06	3.46 ± 0.09

### 3.2 Emission Factors

**Figure 2:**
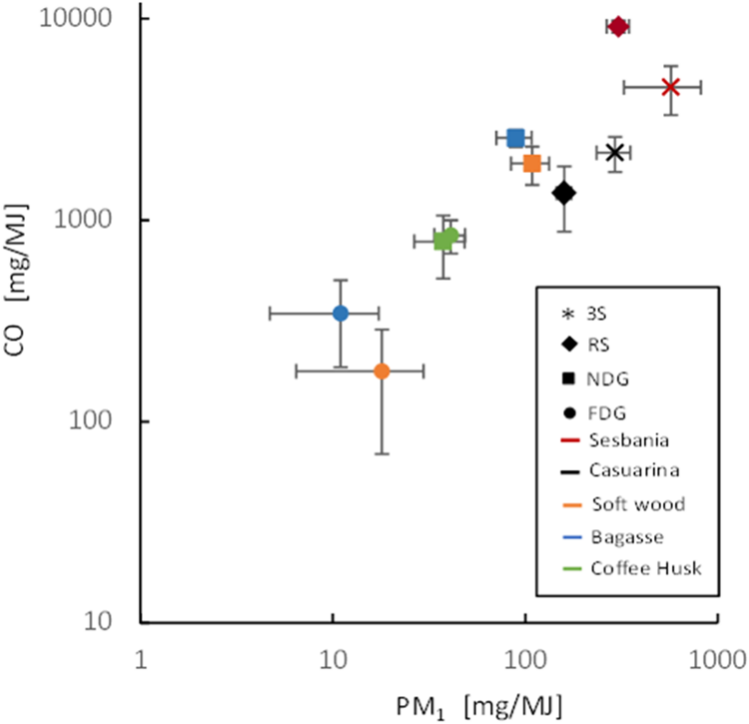
Emissions of PM_1_ and CO (mg/MJ fuel supplied) for the 10 stove–fuel combinations, shown as average values (3 replicates) with standard deviations as horizontal and vertical bars. The different stoves tested were three-stone open fire (3S), rocket stove (RS), natural draft gasifier stove (NDG), and forced draft gasifier stove (FDG).

Emission factors were calculated and presented as milligrams or μg of the specific component per MJ energy supplied with the fuel, determined from an average of three replicate experiments for each stove and fuel combination. The emission factors for gas species, i.e., CO, methane, and BTX, as well as for PM are summarized in Table 3. As described, the results on PM emissions are, in this study, assumed to correspond to PM_1_. In general, a considerable variation (range) in emission factors was seen for different pollutants, not at least for CO and PM_1_ as shown in Figure 2. There is a general trend of reduced emissions with increased stove technological level, as has been well documented.^33,35,42^ Further, it was seen that the emission variation within stove–fuel combinations decreased with an increased technology level. The lowest gaseous and particulate emissions were observed for the FDG, which was fueled with bagasse. However, the present emission and combustion performance results (Section 3.1) also show some intricated variations for specific stoves when using different fuels. The importance of assessing these complex relations has previously also been addressed.^26,42^

**Table 3:** Average Gaseous Emissions, PM_1_ Emissions, and Chemical Particle Characteristics for Each Stove and Fuel Combination

	three stone (3S)			rocket stove (RS)		natural draft gasifier (NDG)			forced draft gasifier (FDG)		
	unit	sesbania	casuarina	sesbania	casuarina	softwood	bagasse	coffee husk	softwood	bagasse	coffee husk
CO	[mg/MJ]	4570 ± 1250	2160 ± 430	9140 ± 630	1360 ± 490	1910 ± 410	2560 ± 240	780 ± 270	180 ± 110	340 ± 160	840 ± 160
methane	[mg/MJ]	373 ± 169	139 ± 25	263 ± 37	50 ± 13	93 ± 26	143 ± 28	56 ± 22	4 ± 1	8 ± 4	6 ± 2
aBTX	[mg/MJ]	290 ± 157	108 ± 50	193 ± 75	80 ± 46	174 ± 42	279 ± 16	101 ± 44	68 ± 34	64 ± 32	59 ± 34
BTX/methane ratio		0.8	0.8	0.7	1.6	1.9	2.0	1.8	18	8.0	9.1
PM_1_	[mg/MJ]	571 ± 245	293 ± 58	305 ± 40	159 ± 14	109 ± 24	89 ± 19	38 ± 11	18 ± 12	11 ± 6	41 ± 7
OC/EC		3.16	1.38	1.94	1.14	1.06	2.00	1.28	0.38	1.14	1.10
bSoot	[mg/MJ]	87.3	86.1	32.5	46.9	40.1	20.6	8.24	10.5	3.42	4.06
cOM	[mg/MJ]	426	183	97.2	82.6	65.5	63.6	16.3	6.15	6.00	6.90
dPAC	[μg/MJ]	953	560	317	133	319	416	145	70	59	48
p-PAH	[μg/MJ]	834	465	250	102	248	309	86	58	44	33
O-PAH	[μg/MJ]	47	41	42	17	36	40	20	9.3	10	8.2
a-PAH	[μg/MJ]	44	5.3	4.8	0.52	4.6	5.1	1.0	0.03	0.01	0.01
PANH	[μg/MJ]	28	49	20	13	31	63	39	1.9	4.5	6.7
PAC/OM	[%]	0.22	0.3	0.26	0.16	0.48	0.64	0.78	1.1	0.95	0.38
einorganic PM mass	[mg/MJ]	76.2 ± 23.7	26.3 ± 3.12	193 ± 17.3	37.1 ± 10.3	3.56 ± 0.12	6.83 ± 2.15	14.6 ± 8.8	2.32 ± 0.01	2.11 ± 0.36	30.9 ± 0.97

a Sum of benzene, toluene, and xylene.

b Soot is calculated as EC multiplied by a factor of 1.1 to compensate for oxygen and hydrogen in the carbonaceous matrix.^41^

c The organic matter (OM) is calculated as OC multiplied by a factor of 1.7 to compensate for oxygen and hydrogen in different organic compounds.^40,43^

d PAC is the sum of all p-PAH, O-PAH, a-PAH, and PANH, analyzed in this study.

e Inorganic PM mass was defined as major ions, measured by ICP elemental analysis, and compensated with their respective counterions.

For all stove technologies, the present emission levels and variability determined for CO are generally in line with previously reported data for the studied technology groups. In a previous study, average CO emissions in the range of 1600–4200 mg/MJ for the 3-stone open fire, have been reported.^33^ Between four rocket stove types, CO emissions ranged between 1200 and 4200 mg/MJ. The RS-sesbania combination generated a comparably high CO emission factor of 9140 ± 630 mg/MJ which is more in range with the charcoal stoves reported.^33^ It was noted for the RS-sesbania combination that a char bed was built up over time. Thus, a similar phenomenon is seen in charcoal stoves generating elevated CO emissions during smoldering (flameless) conversion of the char. For natural draft gasifier stoves, the present CO emissions were in range with previously reported data.^33^ The CO emissions for FDG seen in the present study (180–840 mg/MJ) are rather low compared to previously reported data from both lab- (1500–4300 mg/MJ)^33^ and field-based (800–2400 mg/MJ)^34,44^ studies.

The PM emission factors for all of the 3S and RS experiments were comparable (293–571 mg/MJ and 159–300 mg/MJ, respectively) in this study compared to previously reported data for 3-stone open fire and rocket stoves (150–500 and 80–250 mg/MJ, respectively).^33^ For both stoves, casuarina generated lower emission factors than sesbania. For the NDG stove, there is no significant difference in PM emission factors between the present and previous studies.^33^ However, significantly higher CO and PM emission factors were seen for both softwood and bagasse compared to coffee husk. This was likely an effect of air starvation in the flame burnout phase when the NDG stove combined with these fuels was used due to their higher combustion rate. This phenomenon occurs when the production of pyrolysis gases in the fuel bed, generated by heat from the partial combustion induced by the primary air, exceeds the oxidation capacity of the secondary air. Further, the present PM emission factors for the FDG stove (10–40 mg/MJ) were similar, in the lower range compared with previously reported data (13–254 mg/MJ).^32,33,36^ Opposite to the NDG stove, significantly higher emission factors for both CO and PM were observed when coffee husk was used in the FDG compared to those in the other fuels. Thus, the emission data on both CO and PM for the forced draft gasifier in general illustrates that the stove design, fuel properties, and operation can significantly impact the emission performance for this type of advanced stove technology.

**Figure 3:**
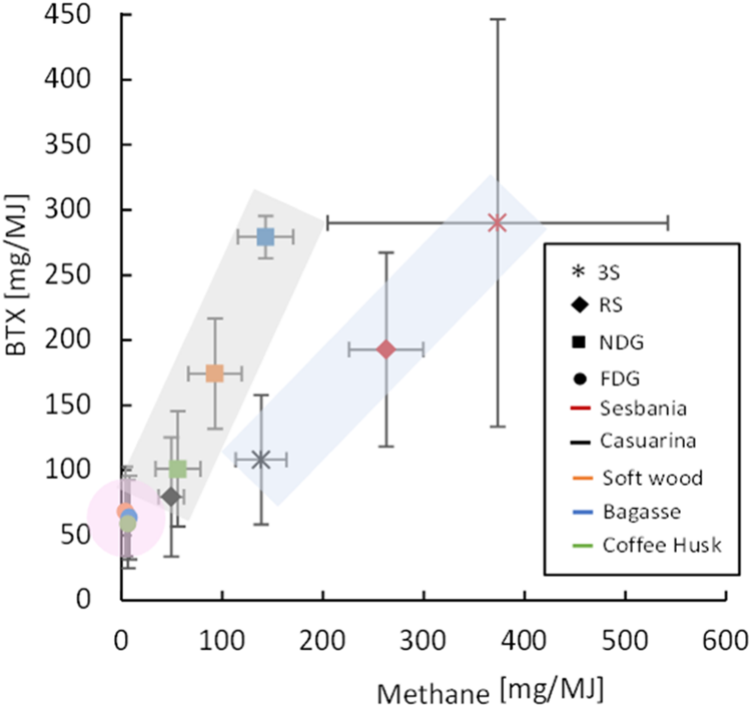
Emissions of methane and BTX (as mg/MJ fuel supplied) for the 10 stove–fuel combinations are shown as average values (3 replicates) with standard deviations as horizontal and vertical bars. The three different indicative BTX/methane ratio areas discussed here are marked by shaded areas; (i) ratio 0.7–0.8 (blue), (ii) ratio 1.6–2.0 (gray), and (iii) ratio 8.0–18 (pink). The different stoves tested were 3-stone open fire (3S), rocket stove (RS), natural draft gasifier stove (NDG), and forced draft gasifier stove (FDG).

The interactions between the stove and fuel were also observed to influence methane and BTX emissions, as shown in Figure 3. The highest emissions of methane were emitted when sesbania was used in both the 3S and RS. By looking at the BTX/methane ratio, a draft distinction of three groups can be defined: (i) ratio: 0.7–0.8 (3S-sesbania, 3S-casuarina, and RS-sesbania), (ii) ratio: 1.6–2.0 (RS-casuarina and NDG with all fuels), and (iii) ratio: 8.0–18 (FDG with all fuels). A presumable cause for the differences in this ratio may be related to different formation pathways and thermochemical conversion behavior of BTX and methane during combustion. While methane starts to form already in the primary pyrolysis of the wood, primarily through demethylation of lignin, BTX are mainly secondary pyrolysis products formed in the flame.^45,46^ In addition, it has previously been established that secondary pyrolysis products are in general less susceptible to oxidation than primary pyrolysis products.^47^ This suggests that the relatively high methane emissions for the first group were caused by methane generated in the primary pyrolysis step bypassing the flame, thus not finally oxidized and combusted.

For the second group with relatively high BTX emissions, the primary pyrolysis products pass through the flame being heated and thermally degraded into secondary pyrolysis products, although not fully combusted. The relatively high emissions of BTX from the NDG-softwood and bagasse combinations further indicate that the stove became temporarily starved during the peak burn rates. The difference between the two cases with the RS, may be explained by different combustion behavior of the sesbania (lower density) and casuarina (more dense) fuels. Finally, in the third group with a very high BTX/CH_4_ ratio in the FDG stove, the overall organic emissions were relatively low. In this case, BTX which is more thermally stable can pass the combustion zone to a higher degree than methane. Compared to the BTX/CH_4_ ratio reported in previous research^48^ on residential heating stoves, e.g., 0.30–0.35, all results seen in the present study for cookstoves are higher. Overall, due to the limited number of data in this work, combined with potential hidden influences of different fuel- and combustion-related parameters, the discussion about the different trends in BTX/methane ratio should be seen as indicative information that needs to be explored further.

### 3.3 Overall PM Composition

**Figure 4:**
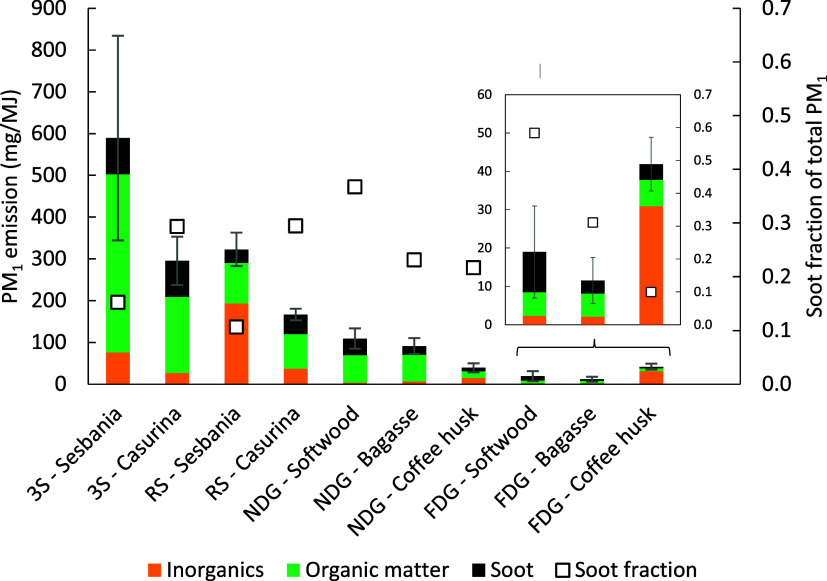
Emissions of PM_1_ (mg/MJ fuel supplied) fractionated into soot (EC * 1.1), organic- (OC * 1.7), and inorganic particulate matter (left axis), as well as the relative soot fraction of the total PM_1_ (right axis), for the 10 stove–fuel combinations, shown as average values (3 replicates) with standard deviations. The different stoves tested were 3-stone open fire (3S), rocket stove (RS), natural draft gasifier stove (NDG), and forced draft gasifier stove (FDG).

The PM_1_ emissions were fractionated into soot (EC * 1.1), organic (OC * 1.7), and inorganic matter by combining the thermal-optical (OC/EC) and ICP analyses (Figure 4). The results show considerabe variations among the different fractions between the stove–fuel combinations. For example, it was seen that although the 3S-casuarina and RS-sesbania combinations generated roughly the same PM_1_ emissions, the composition differed vastly. The inorganic matter constituted more than half of the PM_1_ emissions for the RS-sesbania combination compared to roughly 10% in the 3S-casuarina combination. Furthermore, the combined soot and organic emission factors for the RS-sesbania combination are in the same range as for the RS-casuarina combination. Similarly, the FDG-coffee husk and FDG-bagasse combinations resulted in comparable soot and organic emission factors, although the elevated inorganic content of the FDG-coffee husk combination generated four times higher PM_1_ emission factor. Although coffee husk generated the highest PM_1_ emission factor for the three FDG-fuel combinations, it generated the lowest PM_1_ emission factor in the three NDG-fuel combinations with the largest difference in the emissions of organic matter.

### 3.4 Soot and Elemental Carbon

As discussed previously, it is well known that soot has a decisive role in both the adverse health effects related to air pollution exposure, and climate impacts as one of the most important SLCFs.^12^ The formation of soot in open flames, i.e., turbulent diffusion flames, is very complex and sensitive to several parameters, e.g., temperature, soot precursors concentration, and partial oxygen pressure.^49^ In the flame, primary pyrolysis gases undergo thermal cracking into secondary pyrolysis products and at temperatures above 850–1000 °C further to ternary pyrolysis products, largely forming PAHs and other soot precursors.^29^ A further increase of the heat will further promote coagulation and coalescence of these secondary and tertiary pyrolysis products, primarily PAH, eventually forming soot particles. With increasing temperatures and presence of oxygen, the soot oxidizes and eventually burns up.^46^ Thus, soot formation and oxidation depend on both the stove design and the thermochemical behavior of the fuel. Overall, the results showed that the soot emissions varied by a factor of 25 when comparing the highest soot-emitting stove–fuel combination (87 mg/MJ for 3S-sesbania) with the lowest (3.4 mg/MJ for FDG-bagasse). The soot emissions were very similar for the two 3S-fuel combinations, although the total PM_1_ emissions were a factor of 2 higher for 3S-sesbania compared to 3S-casuarina. A general trend of reduced soot emissions was seen when comparing the 3S with RS, further for NDG, and finally for FDG, showing the lowest soot emissions. However, some interesting exceptions were seen for the NDG-softwood and FDG-softwood combinations, which showed elevated soot emissions compared with when using the other fuels. This may be somewhat surprising since stemwood fuels are generally considered as high-quality “clean” fuels. The elevated soot emissions in these cases are presumably a result of a high degree of fuel volatilization. Subsequently, the stove either lacks sufficient secondary air supply (NDG stove) or provides too short a residence time (FDG stove) to fully oxidize the soot. Furthermore, the NDG showed the highest sensitivity to fuel type with regard to the soot emissions, ranging from 40 mg/MJ using softwood to 21 mg/MJ using bagasse and 8 mg/MJ using coffee husk. The relatively low soot emission seen for the NDG-coffee husk was in the same range as for the FDG-softwood combination with soot emissions of around 10 mg/MJ. The soot constituted between 11 and 59% of the total PM_1_ emissions, with an average between 20 and 30%. The remaining fraction consisted of varying degrees of organic matter and inorganics, depending on the specific conditions for each stove–fuel combination.

### 3.5 Organic Matter

OM consists of several hundred different compounds ranging from larger tar components derived from primary pyrolysis of the fuel that may be further converted to secondary- and tertiary pyrolysis compounds, e.g., PAHs, that have the potential to condense onto existing particles.^4,45^ Similar to soot formation, the cookstove design and the fuel properties will affect the combustion conditions and thus also the formation, conversion, and emission of the OM. The highest OM emission was observed for the 3S-sesbania case, with an average of 426 mg/MJ and constituting 70% of the total PM_1_ emissions. OM emissions were significantly lower (183 mg/MJ) when casuarina was used as fuel. The high amount of OM generated by the 3S was presumably due to primary pyrolysis gases bypassing the flames and, thus, not further oxidized, as previously discussed in Section 3.2. The OM emissions for the RS were even lower when compared to the 3S-casuarina case and in the same range, 82.6–93.7 mg/MJ for casuarina and sesbania, respectively. The enclosed combustion chamber for the RS technology forces the primary pyrolysis gases to pass through the combustion (burnout) zone. For the NDG stove, significantly higher OM emissions were detected for the softwood and bagasse cases compared to the coffee husk case. This further indicates that the NDG stove, when using the two former fuels, became air-starved during the highest burn rates. Due to the high combustion efficiency of the FDG stove, the OM emission factors were rather low for all fuels, in the range of 6–7 mg/MJ, thus, this stove was less sensitive to the combustion behavior of the studied fuels.

From the literature on emissions from residential wood burning, it is well known that OM (OC), composed of primary pyrolysis products of biomass fuel degradation constituents, dominates the PM during slow burning of wood in open fireplaces and stoves. At the same time, soot (EC) formation is more pronounced during higher burn rates and partly air-starved conditions in heating stoves.^50,51^ For biomass cookstoves, it has been shown that the fraction of EC is higher for the improved and advanced stove technologies, especially for the gasifier stoves where the primary pyrolysis gases (high OM) are thermochemically converted in the hot zones of the upper (secondary) combustion sections of the stoves.^52^

### 3.6 Polycyclic Aromatic Compounds (PAC)

**Figure 5:**
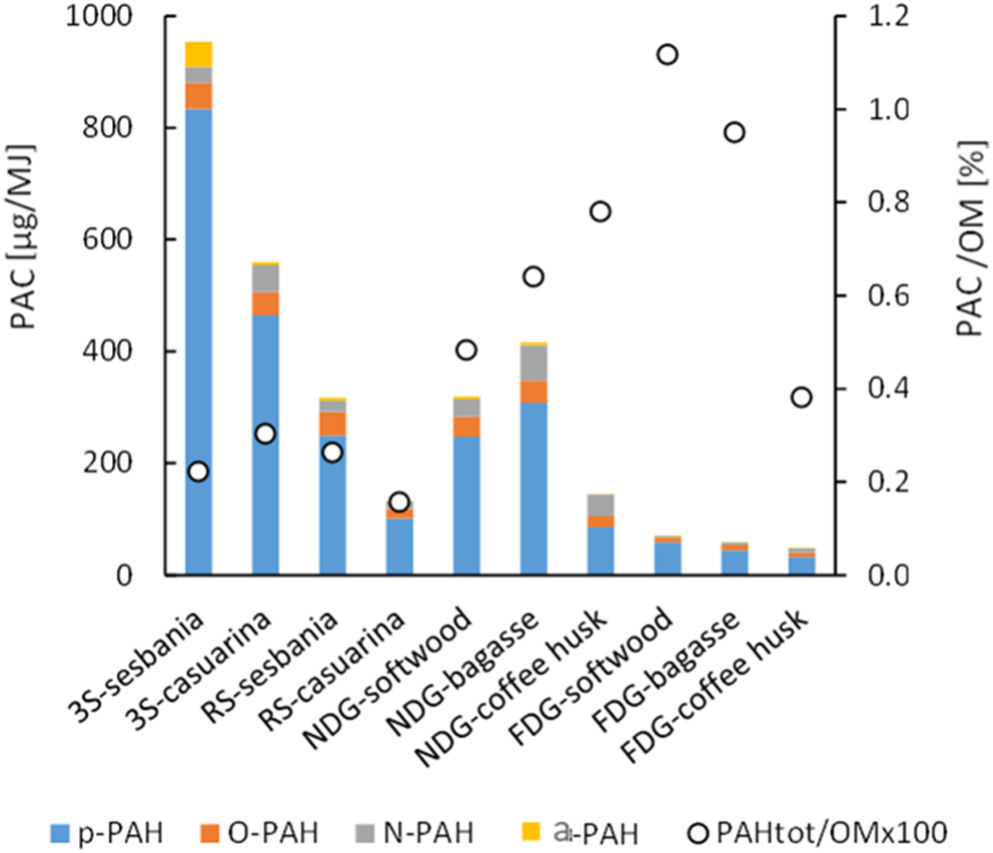
Emissions of total PAC (milligrams per MJ fuel supplied) fractionated into p-PAH, O-PAH, a-PAH, and PANH (left axis) as well as absolute fraction (%) of total PAC of the particulate organic matter (OM) emissions (right axis). The different stoves tested were 3-stone open fire (3S), rocket stove (RS), natural draft gasifier stove (NDG), and forced draft gasifier stove (FDG).

PAC constitutes a wide class of organic compounds with varying and complex structures and the potential to be cancerogenic, including the well-known PAH and its derivatives. PAC are part of the OM, although most often comprising a rather minor fraction of total organics in the PM. As seen in Figure 5, the total PAC emissions in this study varied both between stove technology and fuels used, ranging from 953 μg/MJ for the 3S-sesbania combination to 48 μg/MJ for the FDG-coffee husk combination. In general, the p-PAH composed the largest fraction of the analyzed PAC for all stove–fuel combinations, followed by varying amounts of O-PAH and PANH. In all cases, except for the 3S-sesbania combination, the measured a-PAH emissions were clearly lower compared to the other PAC classes, especially for the FDG stove (0.01–0.03 μg/MJ). Still, the fraction of total PAC emissions in relation to the total amount of OM emitted was lowest for the 3S and RS compared to the gasifier stoves (NDG and FDG).

The PAC emissions from the NDG stove were similar to or even somewhat higher than those from the RS cases when using pelletized softwood and bagasse. These relatively high emissions of PAC from the NDG stove were most likely an effect of the stove becoming air-starved due to high burn rates. The concentration of PAH markers in the PM emissions from a wood stove has been reported to increase dramatically with increased burn rates after a specific load, due to fuel overloading.^53^ In the present study, the high burn rate peaked for NDG fueled with softwood and bagasse, which further supports the argument that the NDG stove became air-starved when using these fuel types.

### 3.7 Inorganic Speciation

**Figure 6:**
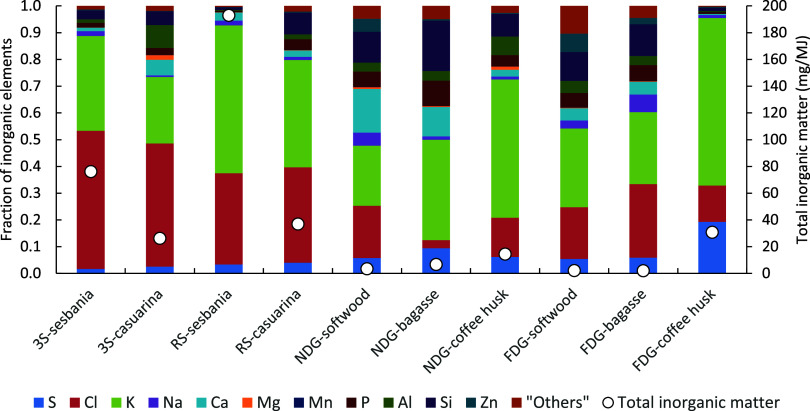
Inorganic elemental distribution, shown as relative fraction (left axis) and total inorganic mass emitted (right axis) for the 10 stove–fuel combinations. The different stoves tested were 3-stone open fire (3S), rocket stove (RS), natural draft gasifier stove (NDG), and forced draft gasifier stove (FDG).

The fractionation into inorganic components based on the results from the ICP-MS analysis shows that the main elements found in the PM were potassium (K), chlorine (Cl), sulfur (S), and in some cases calcium (Ca), as seen in Figure 6. Apart from Ca, these elements together with zinc (Zn) and sodium (Na) are well known to become volatile during biomass combustion and later condense into submicron particles.^32,54,55^ The quantity released of these elements depends on parameters such as the abundance in the fuel, the chemical environment, and the temperature of the fuel particle during combustion. It is, for example, well known that silicon (Si) and phosphorus (P) easily react with Ca, K, and Na, forming different silicates and phosphates in bottom ash, thus reducing the degree of volatilization of alkali (K and Na).^56,57^ By far, the highest emission of inorganic matter was observed for the RS-sesbania case with an emission factor of 193 ± 17 mg/MJ, constituting roughly two-thirds of the total PM_1_ emissions with K and Cl as the main elements. These emissions of inorganic matter were five times higher compared to the RS-casuarina case. Similarly, high emissions of inorganic matter were seen for 3S when using sesbania (76 ± 24 mg/MJ), in this case, three times higher than when fueled with casuarina. For the pelletized fuels in the gasifier stoves, the highest emissions of inorganic matter were seen for coffee husk, for the FDG stove accounting for 75% of the PM_1_. As seen in Table 1, the fuel content of K was twice as high in sesbania compared to casuarina. However, since the increase of inorganic emissions was higher than what can be explained by fuel potassium content only, the results show that the inorganic particle emissions also were influenced by fuel chemistry and combustion temperature.^56,57^ Although more refractory (less volatile) elements such as Ca, Si, and aluminum (Al) are expected to remain in the bottom ashes in stoves and burners, minor levels were found in the PM. Those elements were especially pronounced when the overall inorganic emissions were low, i.e., for the softwood and bagasse cases. This may be explained by traces of coarse refractory particles derived either from fuel ash or through laboratory air via the hood. Overall, the results show that the inorganic fraction, dominated by alkali salts, can have a significant influence of the PM_1_ emissions. Such alkali salts are considered to have relatively low toxicological impact compared to soot and organic matter.^4^ However, the inorganic matter also contains zinc and other trace metals that are more potent to induce toxicological responses.^58,59^

### 3.8 Particle Size Distribution and Number Concentration

The PM mass from all cases was found to have a largely monomodal aerodynamic particle size distribution, in all cases below 1 μm, which confirms the previous assumption that the emissions were dominated by fine (PM_1_) particles. The mass median aerodynamic diameter (MMAD) ranged from 60 to 209 nm for the different stove–fuel combinations. A trend of lower MMAD for the more efficient stoves was seen, with MMAD in the range 60–77 nm for the FDG, in the range 88–97 nm for the NDG, in the range 85–119 nm for the RS, and in the range 138–209 for the 3S. In general, this is all smaller than seen for biomass aerosols generated in residential wood combustion appliances for heating, e.g., wood stoves, and wood pellet boilers.^48,60^

**Figure 7:**
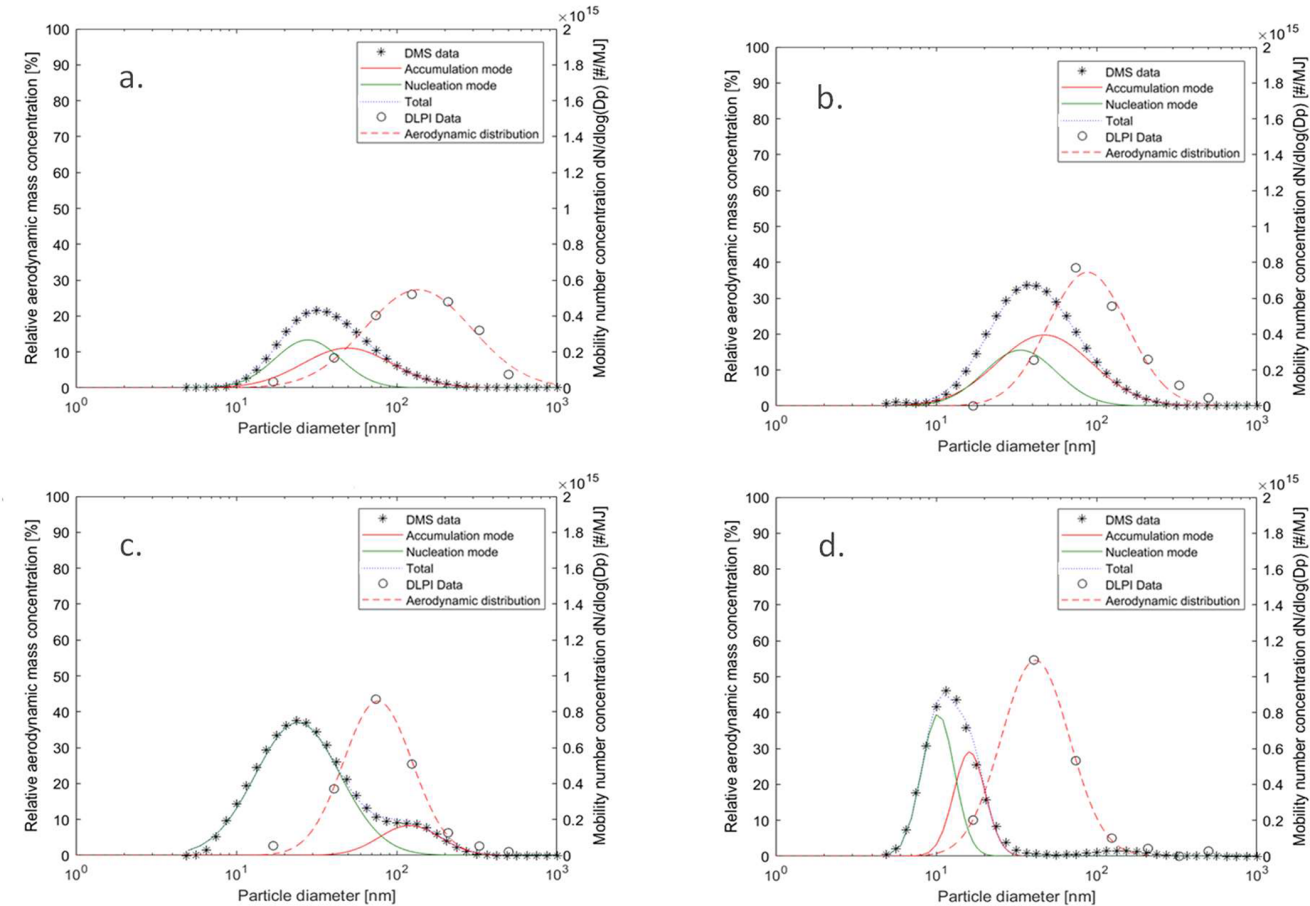
Particle number size distribution (number PSD), mobility-based determined with DMS (“DMS data” illustrated by *) and particle mass size distributions (mass PSD), aerodynamic-based with DLPI (“DLPI data” illustrated by °) from one selected experiment with each of the four stove technologies using woody fuels: (a) 3S-sesbania, (b) RS-sesbania, (c) NDG-softwood, and (d) FDG-softwood. 3S = 3-stone open fire, RS = rocket stove, NDG = natural draft gasifier stove, FDG = forced draft gasifier stove. The number of PSD data was calculated as averages of the scans collected from each complete WBT experiment, while the mass PSD is taken from one impactor sampling for each case. The experimental number PSD data is compared to a fitted bimodal (nucleation- and accumulation mode) log-normal distribution for each case (red, green, and blue curves, where “total” means accumulation + nucleation), and the experimental mass PSD data is also fitted against a monomodal log-normal size distribution (red dotted line).

The mobility-based particle number size distribution (number PSD) data was calculated as averages of the scans collected from a complete WBT experiment (Figure 7). The particle number size distribution data generated by the DMS500 instrument was fitted toward a bimodal (nucleation and accumulation mode) log-normal distribution for each case, to match the experimental data as good as possible. In addition, the data on aerodynamic-based particle mass size distribution (mass PSD) determined by the DLPI, was also fitted against a monomodal log-normal size distribution. Both the experimental size distributions as well as the calculated best-fit size distributions are shown in Figure 7. For the mobility size distributions, it was found that very few particles by number exceeded 300 nm for all stove and fuel combinations. In general, different number size distributions were determined, as seen in Figure 7, with broader distributions for the 3S and RS stoves, a clear bimodal distribution for the NDG stove, and a narrower and largely monomodal distribution for the FDG stove. This dynamics in particle size distributions and shifts in shapes have been illustrated previously for cookstoves,^61^ and are typical for fine biomass combustion aerosols in general, depending on the relative abundance of submicron inorganic-, organic- and soot particles.^4^ However, very little information is available in the present literature about the underlying combustion- and aerosol formation-related explanations governing these observed variations in particle size distributions and dynamics.^61,62^

The count median diameter (CMD) for the so-called nucleation mode varied between 13 ± 1 nm for the FDG-softwood case and 36 ± 4.2 nm for the 3S-sesbania case. Thus, showing a general trend of smaller CMDs for the nucleation mode with improved technology and more complete combustion conditions, in line with previously reported data.^62^ The nucleation mode is expected to be dominated by the inorganic components at higher temperatures near the combustion zone. The increased particle size for the less developed stove technologies is likely caused by condensational particle growth by OM that increases the particle size later in the process as the temperature is reduced in the flue gas channel. Furthermore, no general relationship between stove technology and fuels could be seen with regard to total particle number concentrations per energy used. Only for the FDG stove, it was clear that a higher number of particles and a larger diameter of nucleation mode particles were generated when fueled with coffee husk compared to the other fuels, presumably related to high potassium content and formation of alkali salt particles. However, this trend was not seen for the NDG stove where softwood generated the highest number of nucleation mode particles, and the variations in size were rather small. The OM emissions were considerably higher for softwood and bagasse, which indicates that organic condensation offsets the differences in the particle size of the nucleation mode between the fuels. It was also seen that the particles in the NDG stove generally were slightly larger with a wider distribution compared to the FDG stove. In the NDG-softwood case, it was clear that the soot, in the so-called accumulation mode, also contributed to the number size distribution and increased the general particle size.

For the 3S and RS, the separation of the modes was less clear, presumably due to a more complex aerosol with larger variability over time, as the combustion properties are more variable. To gain more insight, our observations can be combined with a published study (Kristensen et al.) using similar stoves and fuels in a separate campaign.^63^ The fraction of hydrophilic substances (kappa value from size-separated CCNC data) was high for 65 nm (i.e., large fractions of alkali salts), with lower hydrophilicity for the cases with high OM emissions. The hydrophilicity decreased sharply with increasing size, consistent with an increasing fraction of OM and soot. Additionally, the effective density decreased from around 1.2 g/cm^3^ at 65 nm to ∼0.35 g/cm^3^ at 350 nm, with a higher effective density for the OM-rich cases (i.e., 3S). This is consistent with the larger particles above 80–100 nm being soot agglomerates coated to different degrees with OM with only a very small fraction of alkali salts. The high hydrophilicity and high effective density of the smaller particles support that the nucleation mode particles are rich in alkali salts with different degrees of OM coatings and that the soot fraction in this size range is minor. The strongly size-dependent effective density means that the aerodynamic equivalent diameter of the nucleation mode (i.e., alkali salts and organics) and accumulation mode particles (i.e., high fraction of soot) becomes close in size, which implies that they are collected at the same impactor stages. This explains the largely monomodal size-shaped distribution for the impactor measurements in all stove–fuel combinations (see Figure 7).

The measured mobility size distributions are in general smaller than the value of 50–100 nm that has been reported for residential wood heating.^5^ The formation of nucleation particles from alkali salts depends on several parameters, such as cooling rate of the flue gas, dilution factor, and concentration of alkali salts in the flue gas.^64^ One explanation for the relatively small-sized nucleation mode particles seen for biomass cookstoves could be that the hot flue gases travel a short time from the combustion zone before they reach the pot where they are rapidly cooled, quenched, and diluted. Thus, the growth of both the nucleation- and accumulation mode particles are inhibited compared with the processes in residential wood stoves and boilers, where the flue gases are less diluted and slowly cooled in the appliances and chimneys, enabling the growth of the nucleation mode.^31,32,48^ The interpretation of the size distribution data on biomass cookstoves in this study and a comparison with similar data on residential biomass heating appliances can be supported by data on particle number concentrations. The particle number emission factors determined in the present study were in all cases relatively similar between all stove–fuel combinations, ranging (2–6) × 10^14^ #/MJ. For the FDG stove, the particle number emission factors were found in the lower region of this range as compared to the other stoves.

Limited information about particle number emission factors from biomass cookstoves has been reported. In one study, the emission performance during different operational- and stove design modes for a forced draft gasifier stove using hardwood pellets, was studied in laboratory conditions, with particle number emission factors around (2.6–3.2) × 10^14^ #/MJ.^67^ Furthermore, when comparing with the more extensive data available on particle emissions from residential biomass heating appliances, the results on particle number emission factors determined in the present study, are in all cases higher as compared to typical Nordic wood heating stoves ((1.3–4.2) × 10^13^ #/MJ),^48^ conventional masonry wood heaters (7.7 × 10^13^–2.1 × 10^14^ #/MJ, recalculated using a net heating value of 18.3 MJ/kg),^65^ residential wood pellet stoves (4.0 × 10^13^–2.5 × 10^14^ #/MJ), and small-scale boilers using different biomass fuels ((1.6–9.5) × 10^13^ #/MJ).^55^ This may be explained by the influence of flame quenching on cold surfaces of the pot in cooking applications, leading to either elevated nucleation processes or reduced particle coagulation within the flame and flue gases.

Furthermore, in a study by Löndahl et al, it was shown that the particle size distribution from fresh hygroscopic biomass smoke was shifted up from around 80 nm at dry conditions to over 300 nm at the high humidity in the lung due to hygroscopic growth, and in turn, reduced the deposition fraction by almost a factor of 3.^66^ Considering the FDG stove, burning coffee husk where the majority of the particles are considered ultrafine particles (<100 nm) consisting mainly of hygroscopic potassium salts the overall health impact would likely be lower than if the particle emissions, as today, are generalized into only particle mass. In addition, when comparing the aerodynamic and mobility size distributions for the case NDG fueled with softwood (Figure 7c), the aerodynamic mode is seen between the two mobility modes, i.e., around 100 nm. Thus, applying a cyclone or an impactor for particle mass sampling or predefining the ultrafine mass fraction (PM_0.1_) before subsequent number sizing would imply that the aerodynamic mode in this case would be cut in half, while the mobility distribution would be highly undefined. Accordingly, the questions related to what PM fractions and properties actually are measured through aerodynamic quantification of the ultrafine fraction of the PM emissions needs further considerations, not at least in light of the highly variable properties of the particulate emissions seen for different cookstove technologies and fuels used.

## 4 Conclusions

In this work, the emission performance of four cookstoves with different technological advancements was investigated using, in total, five different biomass fuels. The influence of stove technology and fuel interactions on particle emissions and its detailed properties was explored. Up to a 50-fold reduction of PM emissions (all below PM_1_) could be achieved by transitioning from the traditional 3-stone open fire to higher technological stoves in combination with appropriate fuels. Apart from the immense reduction potential in emissions when improved or advanced stoves were introduced, it was also shown that the variability in emission performance for each stove technology decreased with stove technological advancement. This indicates that the combustion conditions became both improved and more consistent. Our findings also show that the fuel type has a significant role in specific PM emission characteristics, with special regard to PACs and inorganic components. It was further seen that both the aerodynamic- and the mobility size distributions were shifted toward smaller particle sizes with higher technological stove advancement. The most important findings and outcomes from this study can be summarized as follows:

- A general trend with a reduction in PM_1_ emissions could be seen with increased cookstove technological advancement, with significant reduction in OM and BC. However, nonlinear influences between cookstoves and fuels were seen regarding other specific particle properties.
- An increase in cookstove technology advancement, in combination with upgraded fuels like pellets, has the potential to enable high combustion efficiency with a broader biomass feedstock, i.e., utilization of different forestry- and agriculture-based fuels.
- Fuel properties, both related to organic (combustible) matrix and inorganic (ash) content, influence the overall combustion and emission performance, specifically the fine (<1 μm) particle physical- and chemical properties.
- Bimodal particle mobility number size distributions were seen for the gasifier stoves, especially for the NDG, while the aerodynamic mass size distributions in general were monomodal (MMD between 65 and 97 nm). Thus, defining ultrafine (<100 nm) PM fractions based on aerodynamic size, risks entailing an uncertain and irrelevant classification with regard to the particle number size distribution.
- The conditions in the combustion zone within the stove play a crucial role when it comes to the formation of specific PM components like OM, BC, and PAC. Especially the emissions of PAC seem to be particularly sensitive to air starvation, thus careful design of the combustion zone is needed to balance with the rate of devolatilization for a particular fuel.
- The stove and fuel combination affects the fuel bed temperature, which influences the formation of inorganic PM, potentially impacting both total PM_1_ emissions and number concentrations. Technological advancements, with higher combustion efficiencies, can imply a shift toward smaller particle sizes, still dominated by alkali salts.

Overall, it was illustrated that the influence of fuel properties (e.g., combustibility, ash content, and composition) and combustion conditions in the stoves (e.g., air supply and distribution and fuel bed temperature) are very complex, and no clear correlations were seen between the stove technologies and fuels related to particle properties. Thus, the combustion of a specific fuel that generates relatively low emissions in one stove might not be as suitable in another stove. Furthermore, some phenomena were seen, such as high PACs and soot emissions due to air starvation, but also elevated inorganic PM emissions linked to fuel ash behavior, combustion efficiency, and temperature. These phenomena have been extensively studied for biomass combustion and aerosol formation in other bioenergy sectors but less related to household cooking with biomass. Unlike in medium- and large-scale biomass combustion appliances, it is difficult to apply advanced appliance design, process control, and flue gas cleaning systems that compensate for potential variability in fuel and combustion conditions in small cookstoves. However, a broad variability of stove technology designs exists with a potential also to utilize a broader range of biomass fuels. Thus, the need for a methodology to classify and “grade” fuels with regard to their combustion properties is emphasized. Likewise, the stoves should be tested to define which fuel category is most suitable for each stove. Furthermore, the high concentrations of inorganic PM (i.e., mainly alkali salts) for sesbania and coffee husk imply difficulties in assessing the emission performance if only particle mass concentration and not chemical composition is considered. For example, the 3S-casuarina case generated the same particle mass emissions as the RS-sesbania case, although with vastly different particle compositions. Finally, this study has clearly illustrated the considerable variation in particle emissions and properties related to specific influences of both biomass fuel properties and cookstove design/technological level. Thus, the need for improved testing and assessment procedures is emphasized to support the development and implementation of future bioenergy and clean cooking strategies.
